# Targeting Adipocyte Enhancer-Binding Protein 1 to Induce Microglial Phenotype Shift for Immunotherapy in Alzheimer’s Disease

**DOI:** 10.3390/ijms27010296

**Published:** 2025-12-27

**Authors:** Eun-Ji Kim, Byeong-Hyeon Kim, Ye-Bin Mun, Minho Moon, Pyung-Hwan Kim

**Affiliations:** 1 Department of Biomedical Laboratory Science, Konyang University, Daejeon 35365, Republic of Korea; kimej1910@gmail.com (E.-J.K.); choel1216@naver.com (Y.-B.M.); 2 Department of Biochemistry, College of Medicine, Konyang University, Daejeon 35365, Republic of Korea; qudgus1250@naver.com; 3 Research Institute for Dementia Science, Konyang University, Daejeon 35365, Republic of Korea

**Keywords:** neuroinflammation, microglia, adipocyte enhancer-binding protein 1, neuronal damage

## Abstract

Neuroinflammation, a key contributor to neurodegenerative diseases, results from excessive microglial activation. Microglia that respond to pathogenic molecules switch to the M1 type and secrete various immune cytokines, which can cause neuronal damage. Therefore, our study focused on molecules that can enhance the neuroprotective role of microglia and reduce neuronal damage. The adipocyte enhancer-binding protein 1 (AEBP1) gene is known for its role in regulating immune responses in macrophages. However, its role in neuroinflammation has not been fully explored. Therefore, we investigated the role of AEBP1 in microglial cells activated by lipopolysaccharide (LPS). First, we confirmed that AEBP1 is expressed in LPS-activated microglia and demonstrated that downregulation of AEBP1 using shRNA in activated microglia reduced the immune response via the nuclear factor-kappa-B (NFκB) pathway. These results promote a shift toward neuroprotective M2 microglia, thereby reducing neuronal damage. Next, we confirmed that the expression of AEBP1 was elevated in the brains of Alzheimer’s disease (AD) mice. Additionally, animal experiments to assess the therapeutic effects of AEBP1 showed that microglia gathered around amyloid beta (Aβ) and reduced its size. Taken together, our results provide the first evidence that AEBP1 can reduce inflammatory activity in microglia, suggesting its potential as a target molecule for immunotherapy.

## 1. Introduction

Neurodegenerative diseases refer to diseases with functional changes, such as aggregation of native proteins, gradual degeneration of nerve cells, behavior, cognitive/executive functioning, or motor dysfunction, including Alzheimer’s disease (AD), Parkinson’s disease (PD), and Huntington’s disease (HD) [1]. It is known that each neurodegenerative disease is caused by various factors, such as genetic and environmental factors. What these diseases have in common is the activation of the brain’s immune response, causing neuroinflammation [2]. For the most studied AD examples, the treatments developed and prescribed to patients use drugs that slow the progression of the disease, such as acetylcholinesterase inhibitors (e.g., donepezil and galantamine) or N-methyl-D-aspartate (NMDA) receptor antagonists. Recently, Lecanemab, a drug that directly targets amyloid beta (Aβ), has been developed as an anti-amyloid treatment, but it has not been widely commercialized due to its side effects and the fact that it can only be applied to early-stage patients [3,4,5,6]. Although many approaches to the treatment of neurodegenerative diseases target the cause of the disease, these treatments show many unstable factors, requiring treatment with other methods. This led us to focus our research on utilizing components of the body’s immune system or their derivatives to combat the causative agents of various neurodegenerative diseases through immunotherapy [7,8].

Neuroinflammation, which is known to cause neurodegenerative diseases, is mainly caused by immune cells such as microglia and astrocytes. In the case of AD, tau and Aβ accumulation induce the activation of immune cells. Tau protein is hyperphosphorylated and forms neurofibrillary tangles with harmful effects [9], while Aβ is produced when amyloid precursor protein (APP) is cleaved by β-secretase (BACE1) and acts as a neurotoxin [10]. These proteins produced in this way act as damage-associated molecular patterns (DAMPs) that bind to receptors such as toll-like receptors (TLRs) and receptors for advanced glycation end products (RAGE). This binding triggers the secretion of cytokines and chemokines, leading to the recruitment of peripheral glial cells. Initially, activated microglia phagocytose the misfolded proteins. However, as the disease progresses, microglia no longer phagocytose misfolded proteins and instead amplify immune responses, leading to neurotoxicity [11,12,13].

Microglia, the main cause of neuroinflammation, differentiate into two types depending on the substance that induces their activity. When microglia are activated by lipopolysaccharide (LPS) or interferon-γ (IFN-γ), signals are transmitted through Toll-like receptor 4 (TLR4) and IFN-γ receptor, respectively, activating inflammation-related signaling pathways, including nuclear factor-kappa-B (NFκB). This polarizes them into the M1 phenotype, which secretes inflammatory cytokines, such as IL-6 and TNF-α. In contrast, microglia activated by IL-4 or IL-13 activate the JAK-STAT signaling pathway, particularly the STAT3 or STAT6 pathway, through the IL-4 receptor/IL-13 receptor, and secrete cytokines that promote anti-inflammatory responses or polarize them into the M2 phenotype, which increases the expression of phagocytic receptors such as TREM2 and SR-A1, and participates in the clearance of misfolded proteins such as Aβ [14,15,16,17]. Due to these characteristics of microglia, there is increasing interest in substances that can shift activated M1 microglia toward the M2 phenotype as a potential therapeutic approach for treating neurodegenerative diseases. Therapeutic development strategies for AD are being explored in the direction of regulating microglial cells to address the underlying issues.

To address this point, we focus on adipocyte enhancer-binding protein 1 (AEBP1), a protein associated with immune responses. AEBP1 is a transcriptional repressor with carboxypeptidase activity [18]. It has been reported to contribute to the maintenance of cholesterol homeostasis in macrophages, as well as to regulate immune responses via the NF-κB pathway [19,20,21]. A recent brain genomic analysis of AD reported that the *AEBP1* gene is upregulated in microglia [22]. The expression of AEBP1 was also reported to be increased in the hippocampus among the brain tissues of patients with AD [23]. These previous studies suggest that AEBP1 may play a role in immune responses under inflammatory and pathological conditions.

Given the functional similarities between macrophages and microglia as immune cells, the reported role of AEBP1 in macrophages suggests its possible involvement in regulating inflammatory responses in activated microglia. However, studies on the biological function of AEBP1 in microglia are still limited; therefore, we aimed to elucidate the role of AEBP1 in microglia under inflammatory stimulation conditions.

Overall, in this study, we confirmed whether AEBP1 was expressed in activated microglial cells and evaluated how the regulation of its expression affects neurons in vitro. In addition, using an Alzheimer’s disease mouse model, we analyzed the effect of regulating AEBP1 expression on microglial function in the diseased brain environment.

## 2. Results

### 2.1. The Optimal Condition of LPS Treatment for the Induction of AEBP1 Expression in Microglial Cells

**Figure 1:**
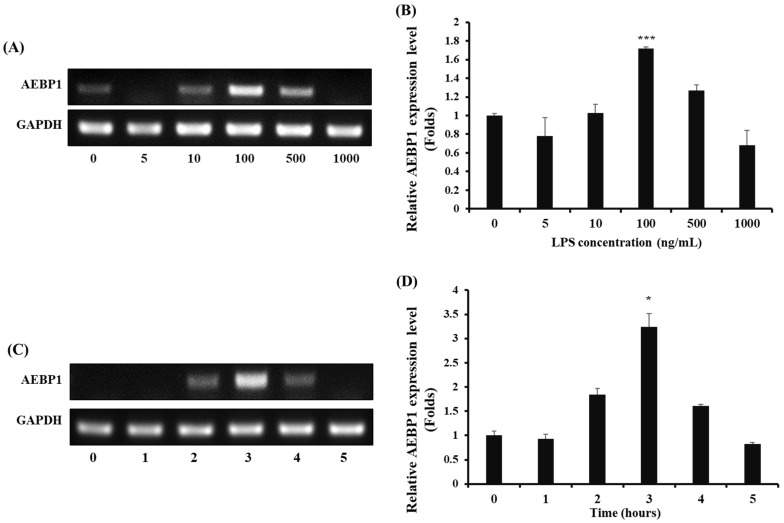
The optimal LPS conditions for inducing AEBP1 expression in BV2 microglial cells. (**A**) AEBP1 expression depending on various concentrations of LPS and (**B**) Quantified values of AEBP1 expression induced according to LPS treatment concentration. (**C**) AEBP1 expression at various time points of treatment with 100 ng/mL LPS and (**D**) Quantified values of AEBP1 expression depending on the 100 ng/mL LPS treatment time. The y-axis “fold” values represent densitometric quantification normalized to the loading control (GAPDH) and expressed relative to the control BV2 group (set to 1). Data are presented as mean ± SD (*n* = 3). * *p* < 0.05, *** *p* < 0.01 compared with untreated BV2 cells (0 ng/mL).

To activate microglial cells, LPS was used as a stimulant, and the expression of AEBP1 was assessed in microglial cells treated with LPS. First, BV2 microglial cells were treated with various concentrations and treatment times of LPS to determine the conditions that induced AEBP1 expression. As shown in Figure 1A,B, AEBP1 expression significantly increased when BV2 cells were treated with a 100 ng/mL concentration of LPS. Next, we determined the optimal treatment time for AEBP1 expression in activated microglial cells. BV2 cells were treated with 100 ng/mL LPS for varying time periods, and the expression of AEBP1 was evaluated. It was observed that the highest expression of AEBP1 occurred in BV2 cells treated with LPS for 3 h (Figure 1C,D). Therefore, treatment of BV2 microglial cells with LPS at a concentration of 100 ng/mL for 3 h was confirmed to induce maximal AEBP1 expression. This result indicates that AEBP1 is expressed in activated microglia.

### 2.2. Selection of the Best shRNA for the Downregulation of AEBP1 in Activated Microglial Cells

**Figure 2:**
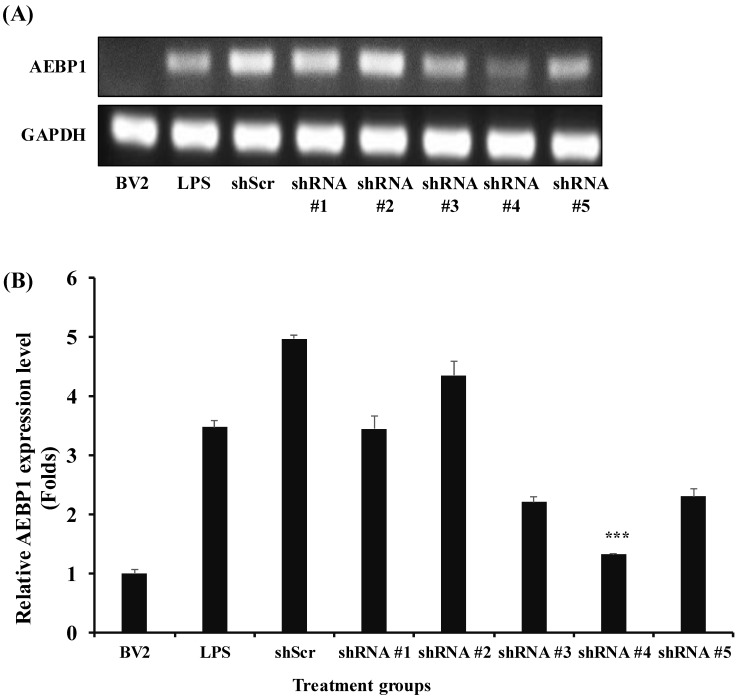
Selection of shRNA for the best downregulation of AEBP1 in activated BV2 microglial cells treated with LPS. (**A**) The decision of shRNA showing the best knockdown efficiency using conventional PCR. (**B**) Quantified values relative to GAPDH expression. The y-axis “fold” values represent densitometric quantification normalized to the loading control (GAPDH) and then expressed relative to the control BV2 group (set to 1). Data are shown as mean ± SD (*n* = 3). *** *p* < 0.01 compared with the LPS-treated group.

Based on the optimal conditions selected from the results shown in Figure 1, we confirmed that AEBP1 was expressed in microglial cells activated by LPS. To evaluate the biological function of AEBP1 in microglial cells, a series of shRNAs capable of knocking down this gene was designed and used for treatment. The results shown in Figure 2 indicate that shRNA #4 induced a more significant downregulation of AEBP1 than the other shRNA series. In the following experiments, shRNA #4, referred to hereafter as shAEBP1, was used to induce the downregulation of AEBP1.

### 2.3. The Decreased Immune Function of the Activated Microglial Cells by the Downregulation of AEBP1

**Figure 3:**
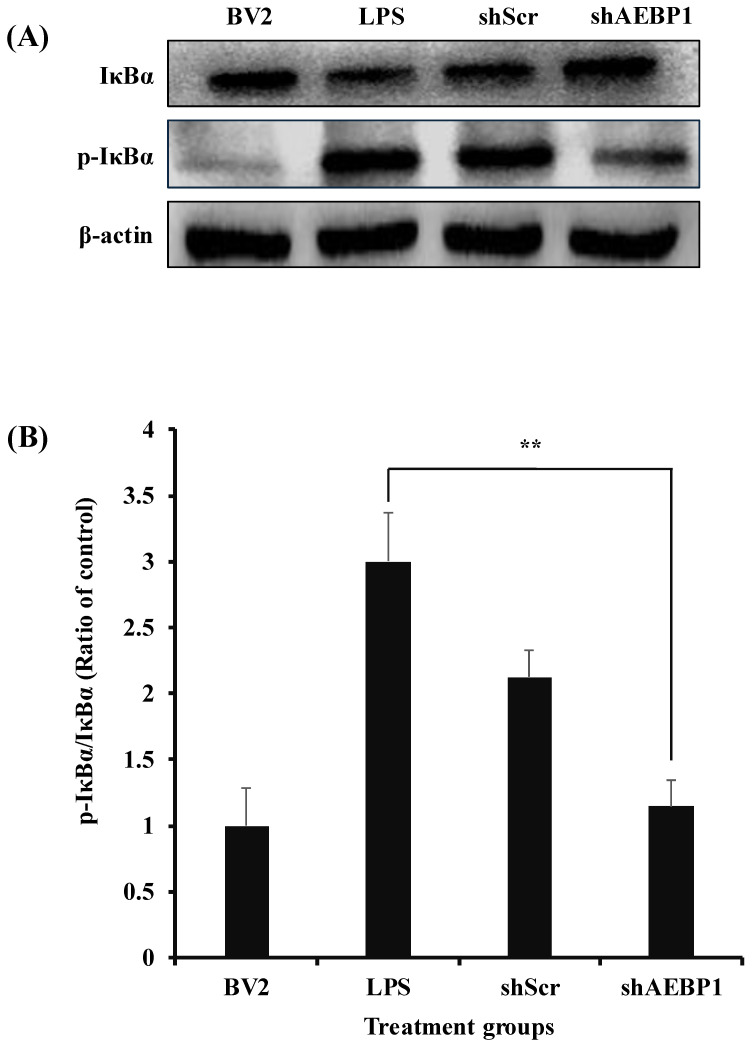
The decreased I*κ*B*α* phosphorylation by AEBP1 knockdown in BV2 microglial cells. (**A**) The expression level of p-IκB*α* in BV2 cells treated with shAEBP1 evaluated by western blotting. (**B**) Quantitative values of p-IκB*α* by western blotting. Data are shown as mean ± SD (*n* = 3). ** *p* < 0.02 compared with the LPS-treated group.

The NFκB pathway plays a crucial role as a mediator in immune responses. When NFκB is activated, it induces the expression of pro-inflammatory genes, thereby triggering an inflammatory response. NFκB responds to pathogens and can phosphorylate IκBα to initiate immune-related reactions [24,25]. *AEBP1*, known as an NFκB modulator in macrophages, has been shown to promote the phosphorylation of IκBα, thereby inducing the activation of the NFκB pathway [21,26]. Hence, we investigated changes in the expression of phosphorylated IκBα (p-IκBα) upon downregulation of AEBP1 in activated microglial cells. As shown in Figure 3, LPS-treated BV2 cells showed a significantly increased expression of p-IκBα protein. In contrast, BV2 cells treated with shAEBP1 exhibited decreased expression of p-IκBα protein compared with those in the LPS or shScr group. This result implies that the downregulation of AEBP1 in activated BV2 cells suppresses the phosphorylation of IκBα, a known negative regulator of the NFκB pathway.

**Figure 4:**
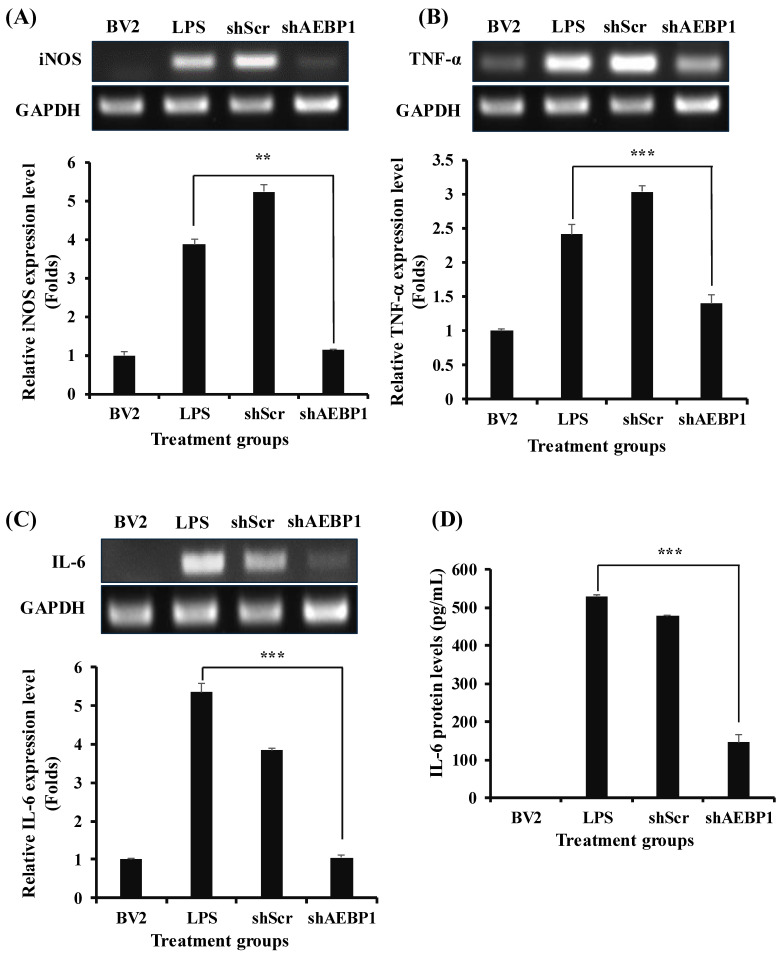
Decreased cytokine expression by AEBP1 knockdown in BV2 microglial cells. Immune cytokine expression and quantified values in AEBP1-downregulated BV2 microglial cells for (**A**) iNOS, (**B**) TNF-α, and (**C**) IL-6. The y-axis “fold” values represent densitometric quantification normalized to the loading control (GAPDH) and then expressed relative to the control BV2 group (set to 1). (**D**) IL-6 expression levels measured by ELISA. Data are presented as mean ± SD (*n* = 3). ** *p* < 0.02, *** *p* < 0.01 compared with LPS-treated group.

Based on the finding that AEBP1 knockdown affects the NFκB pathway, we evaluated how the expression of immune cytokines secreted by microglia was altered through this mechanism. BV2 cells treated with shAEBP1 showed a significant reduction in the expression of various immune cytokines, iNOS, TNF-α, and IL-6, compared to those treated with LPS or shScr (Figure 4A–C). The shSCR-treated group showed no significant changes compared to the LPS-treated group. This means that shABP1 treatment induces changes in intracellular NF-kB signals.

This tendency was also confirmed for the IL-6 protein level using ELISA (Figure 4D). The results showed a marked decrease in cytokine levels when BV2 cells were treated with shAEBP1. Taken together, Figure 3 and Figure 4 show that the downregulation of AEBP1 can reduce the immune activity of activated microglial cells.

### 2.4. Phenotypic Transition from M1 into M2 of Microglial Cells with the Downregulated AEBP1

When microglia are activated to the M1 phenotype, pathways related to the immune response, such as the NFκB pathway, are activated, and pathways related to the M2 phenotype are suppressed by antagonism [27]. Following the results shown in Figure 3, we used phenotypic markers to verify whether microglia with reduced NF-kB activity due to downregulation of AEBP1 can change their phenotype from M1 to M2.

**Figure 5:**
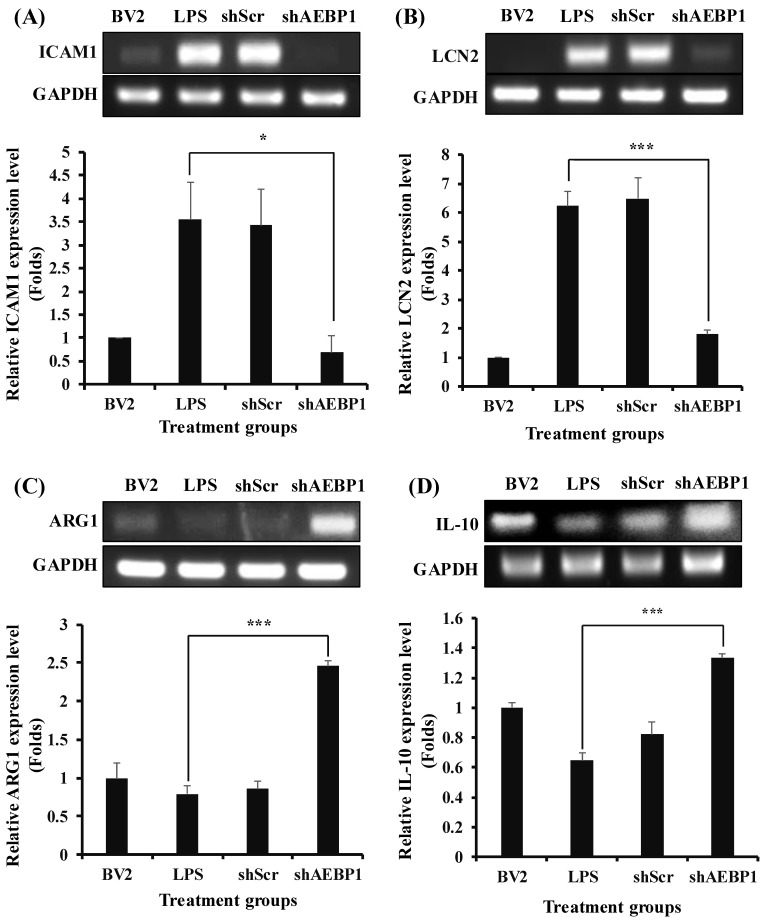
Switching from M1 to M2 phenotype by AEBP1 knockdown in BV2 microglial cells. Transition from M1 to M2 phenotype expressing (**A**,**B**) M1 and (**C**,**D**) M2 markers by downregulation of AEBP1 in activated BV2 cells. The y-axis “fold” values represent densitometric quantification normalized to the loading control (GAPDH) and then expressed relative to the control BV2 group (set to 1). Data are shown as mean ± SD (*n* = 3). * *p* < 0.05, *** *p* < 0.01 compared with LPS-treated group.

To address this, ICAM1 and LCN2 as M1 markers, and Arg1 and IL-10 as M2 markers, were assessed in the activated microglial cells with shAEBP1 to characterize microglial phenotype. As shown in Figure 5A,B, LPS-treated BV2 cells exhibited increased expression of M1 markers compared to the negative control group (untreated BV2 cells). However, when shAEBP1 was used to treat activated microglial cells, the expression of M1 markers significantly decreased to levels similar to those in untreated BV2 cells. In the examination of M2 markers, LPS-treated BV2 cells showed decreased expression, whereas BV2 cells with downregulated AEBP1 exhibited increased expression levels (Figure 5C,D). These results demonstrate that the downregulation of AEBP1 influences the phenotypic transition of microglia, leading to anti-inflammatory function.

### 2.5. The Change of Phagocytic Ability of Microglial Cells Induced by AEBP1 Knockdown

Microglia can phagocytose Aβ or α-synuclein, infection, and damaged neurons [28]. This is important because it can reduce neuroinflammation by removing pathological proteins. This phagocytic ability is associated with the expression of phagocytic receptors on the surface of microglia [29]. These phagocytic receptors are downregulated when microglia exhibit the M1 phenotype due to the secretion of immune cytokines [30]. In contrast, M2 microglia, characterized by lower immune cytokine secretion, are associated with higher expression of phagocytic receptors [16,31]. Therefore, an increase in M2 microglia is expected to enhance phagocytic action against disease-causing molecules, thereby reducing neuronal damage.

To investigate whether AEBP1-downregulated microglia possess the functionality of M2 microglia, we evaluated the expression of receptors known to indicate phagocytic activity in M2-type microglia. Among the many receptors, scavenger receptor class A1 (SR-A1, also known as MSR1), which is known for its high affinity for Aβ [32,33], was assessed for its expression.

**Figure 6:**
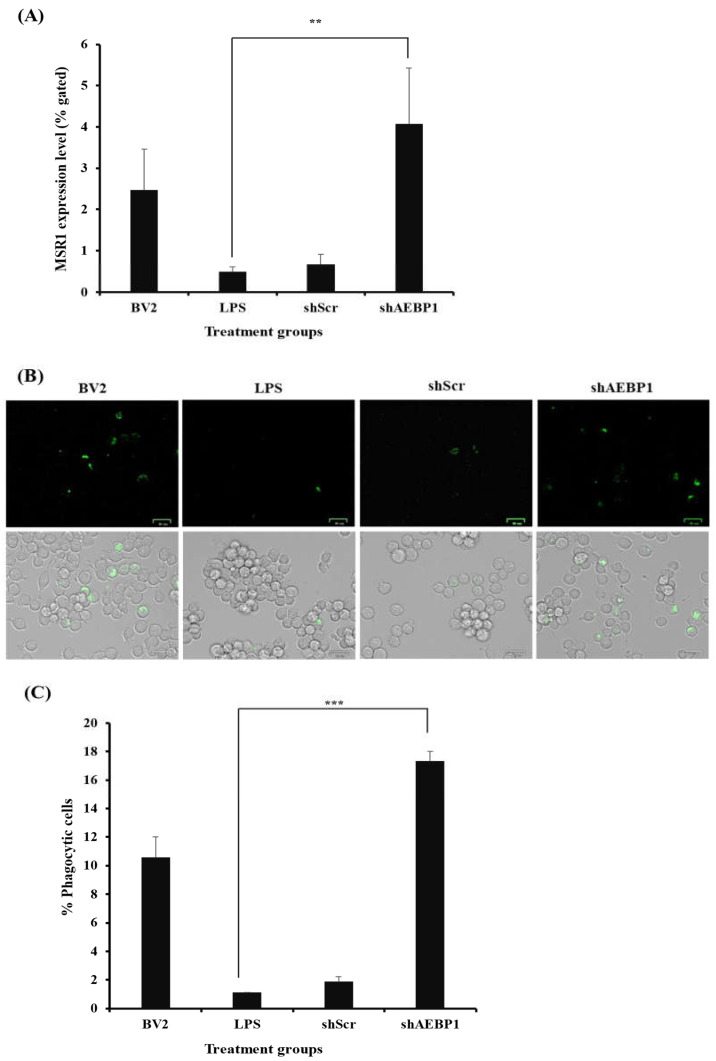
Improved phagocytic activity by AEBP1 knockdown in BV2 microglial cells. (**A**) MSR1 expression in shAEBP1-treated BV2 cells. (**B**) Phagocytic cell images of BV2 cells treated with shAEBP1 using a ZOE fluorescence microscope (scale bar = 25 µm). (**C**) Quantitative graph. Data are presented as mean ± SD (*n* = 3). ** *p* < 0.02, *** *p* < 0.01 compared with LPS-treated group.

First, activated BV2 cells were treated with either shAEBP1 or shScr, followed by incubation with the MSR1 antibody. Results of flow cytometry (Figure 6A) revealed a significant decrease in MSR1 expression in LPS- or shScr-treated BV2 cells. In contrast, AEBP1-downregulated BV2 cells showed the highest expression of MSR1 compared to the other groups. This result demonstrates that the downregulation of AEBP1 not only induces the transition to an M2 microglial phenotype but also enhances the phagocytic activity of microglia.

Subsequently, the phagocytic ability of microglia was evaluated using a phagocytosis kit (Figure 6B,C). BV2 cells were treated with FITC-labeled beads to measure phagocytosis in vitro. When the cells responded to the beads, they exhibited fluorescence. The final results were analyzed by merging the bright and fluorescent field images captured using a ZOE fluorescence microscope. In the results, it was observed that the number of cells exhibiting fluorescence was higher in shAEBP1-treated BV2 cells than in LPS- or shScr-treated cells. As shown in Figure 6, downregulation of AEBP1 in activated microglia increased the expression of phagocytic receptors, resulting in enhanced phagocytic ability of microglia.

### 2.6. The Improved Survival of Neurons Co-Cultured with AEBP1-Downregulated Microglial Cells

**Figure 7:**
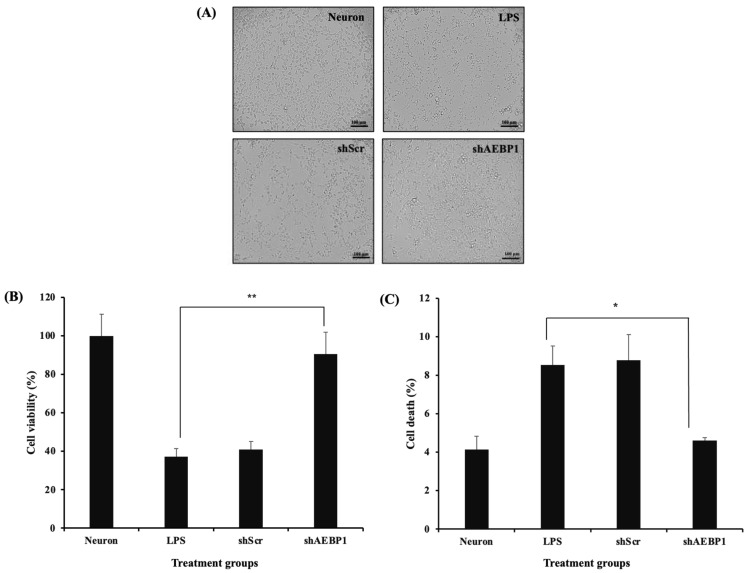
Decreased neurotoxicity of AEBP1-downregulated BV2 microglial cells. (**A**) Bright images of neuron cells co-cultured with shRNA-treated BV2 microglial cells. (**B**) Cell viability as determined by MTT assay. (**C**) Comparison of cell death rate by flow cytometry in a co-culture system with neuron cells and activated microglial cells treated with shRNA. Data are presented as mean ± SD (*n* = 3). * *p* < 0.05 compared with LPS-treated group, ** *p* < 0.02 compared with LPS-treated group.

Recent research has reported that immune factors secreted by activated microglia can induce damage to nerve cells [34]. Therefore, we investigated whether downregulation of AEBP1 in microglial cells plays a neuroprotective role by suppressing immune responses. For this, a co-culture experiment was conducted in which SH-SY5Y cells were seeded in the lower well, and BV2 cells treated with shAEBP1, LPS, or shScr were seeded in the upper well. After 48 h of co-culture, the morphology of SH-SY5Y cells was observed under a microscope (Figure 7A). The morphology of SH-SY5Y cells co-cultured with shAEBP1-treated BV2 cells was similar to that of untreated SH-SY5Y cells, but not to that of LPS-treated microglial cells. The survival rate of neuronal cells co-cultured with shAEBP1-treated BV2 cells was higher than that of cells co-cultured with BV2 cells treated with LPS or shScr (Figure 7B).

To evaluate the mechanisms and cell death rates that led to the results shown in Figure 7A,B, we assessed the apoptotic rate of neuronal cells, which would reflect a decrease in neuronal cell death. Similarly, using a co-culture system, cells were cultured together and then analyzed for apoptosis using flow cytometry (Figure 7C). The results showed that neuronal cells co-cultured with BV2 cells treated with LPS or shScr led to neuronal cell death by increased apoptosis compared to that in neuronal cells alone. Conversely, co-culture of neuronal cells with BV2 cells treated with shAEBP1 resulted in a decreased apoptotic cell population. These results demonstrate that the downregulation of AEBP1 in microglia can reduce neurotoxicity caused by microglia and promote increased neuronal cell survival.

### 2.7. The Expression of AEBP1 in the Mouse Model with AD

We next evaluated the expression of AEBP1 in the brain environment, where pathological proteins accumulate, to assess its potential as a biomarker or therapeutic target for future clinical applications. We attempted to confirm this using a 5× FAD mouse model of Alzheimer’s disease. Genomic DNA was extracted from the frontal cortex (FC), hippocampal formation (HP), and olfactory bulb (OB) tissues, which are known to have abundant microglia in AD model mice (5× FAD) [35,36] and normal wild-type (WT) mice, and the expression of AEBP1 was examined by PCR.

**Figure 8:**
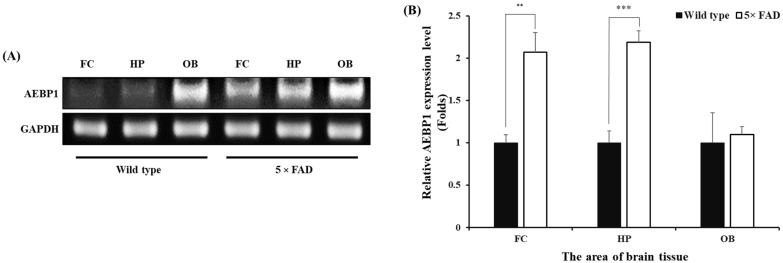
AEBP1 expression level in microglia-rich brain tissues of the Alzheimer’s disease mouse model. (**A**) AEBP1 expression in three brain regions (frontal cortex, hippocampal formation, and olfactory bulb) of 5× FAD mice. (**B**) Quantitative results of AEBP1 expression in brain regions. Data are presented as mean ± SD (*n* = 3). ** *p* < 0.02, *** *p* < 0.01 compared with wild-type mice.

The expression of AEBP1 in brain tissue was higher in AD mice than in WT mice (Figure 8). Specifically, AEBP1 expression in the FC and HP regions of 5× FAD mice was higher than that in WT mice. However, no differences were observed in the OB region. Consequently, the increased expression of AEBP1 in the FC and HP regions of 5× FAD mice indirectly suggests that these regions have more activated microglia than other regions due to Aβ accumulation.

### 2.8. Decreasing the Expansion of Aβ Plaques by Enhancing Microglial Cell Recruitment in the AD Mice Brain Treated with shAEBP1

**Figure 9:**
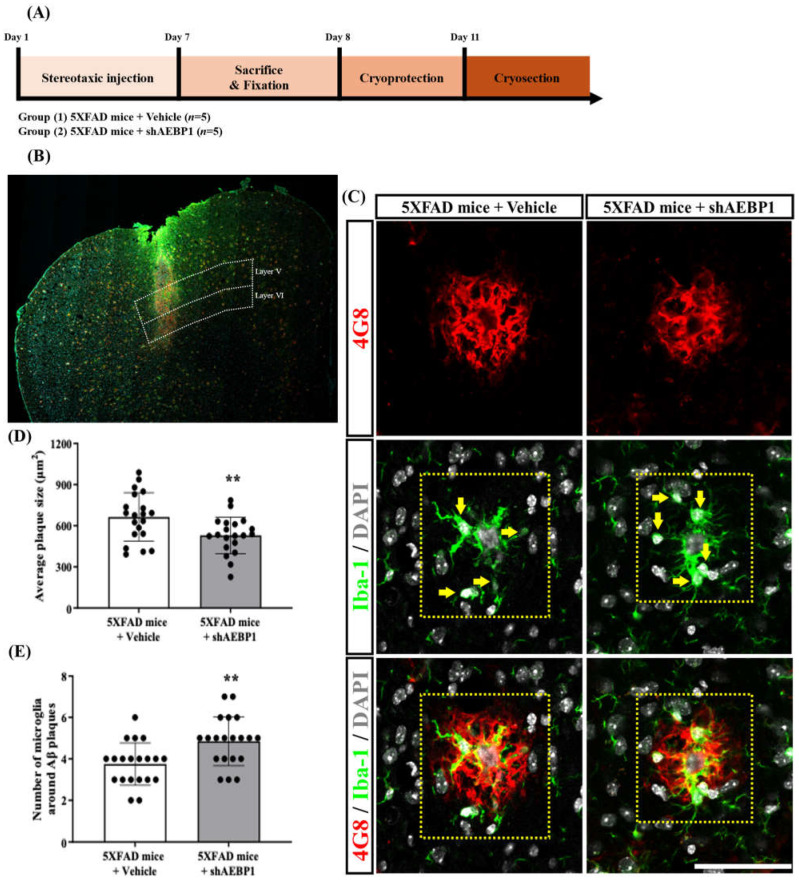
shAEBP1 decreased the expansion of Aβ plaques by enhancing microglial recruitment. (**A**) Schematic workflow of histological analysis to investigate the effect of shAEBP1 on microglial recruitment around the Aβ plaque. (**B**) Representative images of the stereotaxic injection site in layer 5 of the cortex (white box). Scale bar = 500 μm. (**C**) Representative images of 4G8-positive Aβ plaques and Iba1- and DAPI-stained microglia around 4G8-labeled Aβ plaques in layer 5 of the cortex of 5×FAD mice (yellow box). The yellow arrow indicates a microglial cell that is double-labeled with Iba-1 and DAPI. Scale bar = 50 μm. (**D**) The average plaque size was significantly decreased in shAEBP1-treated 5×FAD mice compared to that in vehicle-treated 5×FAD mice. (**E**) The number of Iba-1 and DAPI double-labeled microglial cells around Aβ plaques was significantly increased in shAEBP1-treated 5×FAD mice compared to that in vehicle-treated 5×FAD mice. Values are expressed as mean ± SD. Statistical analysis between the two groups was performed using an independent *t*-test. ** *p* <0.01 indicates significant differences compared with the vehicle-treated 5×FAD mice (white bar).

To determine whether shAEBP1 increases microglial recruitment to Aβ plaques by inhibiting AEBP1, we stereotaxically injected shAEBP1 into cortical layer 5 of 5×FAD mice (Figure 9A,B). The anti-4G8 and anti-Iba-1 antibodies were used to stain Aβ and microglia, respectively. The quantitative values indicated that the average size of Aβ plaques was significantly decreased in shAEBP1-treated 5×FAD mice compared to that in vehicle-treated 5×FAD mice (Figure 9C,D). In addition, the number of Iba-1 and DAPI double-positive microglial cells around Aβ plaques in cortical layer 5 was significantly increased in the shAEBP1-treated 5×FAD mouse group compared with that in the vehicle-treated 5×FAD mice (Figure 9C–E). These results showed that shAEBP1 reduced Aβ deposition in the brain tissues of mice with AD by enhancing microglia recruitment to Aβ plaques.

## 3. Discussion

Neuroinflammation, which causes neurodegenerative diseases, is caused by the overaction of microglial cells that damage nerve cells [15]. Microglia are essentially immune cells that maintain brain homeostasis. Microglia, a type of phagocyte that also functions in the brain, require the activation of various signaling pathways to induce immune responses [27]. A representative example among them is the NFκB pathway. The NFκB pathway is known to be a signaling pathway that is involved in immune responses by inducing the expression of genes related to various immune responses and is also associated with cell proliferation and survival [37].

These microglia exhibit different responses depending on the receptors that bind to foreign antigens. When they bind to receptors, such as toll-like receptors (TLRs) on microglia, they produce a pro-inflammatory response and are characterized as M1 microglia. Conversely, when activated by binding to receptors such as triggering receptor expressed on myeloid cells-2 (TREM2) present in microglial cells, they induce an anti-inflammatory response and appear as M2 microglia [38]. In neurodegenerative brain diseases such as AD, the increased binding of Aβ to TLRs leads to excessive activation of M1-type microglia, resulting in neuroinflammation [16]. For this reason, there is growing interest in methods to reduce the activity of M1 microglia as a therapeutic approach.

In this context, we focused on the *AEBP1* gene to reduce the function of overactivated microglia. AEBP1 is known to enhance cellular immune responses by promoting the phosphorylation of IκBα, leading to activation of NFκB in macrophages [21], and has been reported to be one of the genes expressed in activated microglia through genomic analysis [22]. These findings suggest that the *AEBP1* gene can act as a molecule that induces immune responses in microglia. Indeed, we confirmed that when an external stimulus is applied, activated microglia express AEBP1 in BV2 microglial cells (Figure 1) and evaluated the role of this gene in microglial function. A biological process was observed following treatment with shAEBP1, which inhibited the intrinsic function of this gene. At 3 h, AEBP1 showed a non-monotonic response to LPS. This pattern may reflect dose-dependent kinetics of TLR4/NFκB activation and negative feedback (tolerance), where higher LPS levels can shift the response toward an earlier peak and earlier decline. The downregulation of AEBP1 in activated microglia led to reduced phosphorylation of IκBα (Figure 3), demonstrating that AEBP1 downregulation can inhibit the activation of the NFκB pathway in microglia, similar to the response observed in macrophages. The NFκB pathway is a major signaling pathway involved in the immune response. When activated, the NFκB pathway increases the expression of various immune-related genes and the release of immune cytokines [25,26]. As these immune cytokines cause neuroinflammation, the decreased immune reaction due to AEBP1 downregulation can be predicted to reduce damage to nerve cells. To demonstrate this, we examined the expression levels of immune cytokines secreted by microglial cells by inhibiting NFκB activity (Figure 4). The expression of immune cytokines was reduced in microglial cells with downregulated AEBP1 at both the transcript and protein levels. Through this, we found that AEBP1 suppresses immune responses by regulating the NFκB pathway in microglial cells. Given that inhibition of the NFκB pathway can alleviate symptoms of neuropathy and promote the function of M2 microglia [27], we assessed whether the immune response inhibition observed in microglia with downregulated AEBP1 could transition their phenotype to neuroprotective M2 microglia (Figure 5). The downregulation of AEBP1 in microglia resulted in reduced M1 marker expression and increased M2 marker expression. This suggests that the downregulation of AEBP1 facilitates the transition between microglial phenotypes, potentially promoting their conversion to neuroprotective M2 microglia.

When microglia switch to the M2 phenotype, there is an increased expression of receptors capable of binding to debris and misfolded proteins, leading to enhanced phagocytic ability [39]. These receptors are typically not expressed in environments rich in immune cytokines [29]. Therefore, we evaluated whether M2 microglia induced by AEBP1 could perform phagocytic functions. For this, among the numerous phagocytic receptors, scavenger receptor class A1 (SR-A1), known for its strong ability to bind to Aβ [32,33], was assessed. As shown in Figure 6A, SR-A1 expression increased when AEBP1 was downregulated in microglial cells. These findings indicate that the downregulation of AEBP1 can improve the function of M2 microglia. Based on this result, the phagocytic ability of microglial cells was examined. Figure 6B,C showed that downregulation of AEBP1 can enhance the phagocytic ability of microglial cells, indicating that the downregulation of AEBP1 induces conversion to M2 microglia. Based on the premise that these results would ultimately affect the survival and death of neurons, we evaluated this effect using a co-culture experiment of microglia and neurons. The results shown in Figure 7 indicate that neurons co-cultured with AEBP1-downregulated microglia had increased survival rates. Ultimately, this suggests that the regulation of AEBP1 expression in microglial cells in vitro can act as a substance that protects nerve cells by converting excessively pro-inflammatory reactions into anti-inflammatory reactions.

We next examined the expression of AEBP1 in the brain tissue of AD mice to determine whether this gene has a high potential for clinical application. This was assessed by expression in three regions known to have a high concentration of microglia. The results shown in Figure 8 demonstrate that AEBP1 is expressed in microglia in the brains of mice with AD at the transcriptomic level.

The recruitment of microglia around amyloid plaques in the brains of mice with Alzheimer’s disease suggests that microglia detect and activate mechanisms to remove abnormal substances. Based on our previous results, we confirmed that downregulating AEBP1 in microglia enhances phagocytosis. Based on this, we labeled amyloid plaques and microglia in the frontal cortex with anti-4G8 and anti-Iba-1, respectively. After injecting shAEBP1 into the frontal cortex, we examined the size of amyloid plaques and the number of microglia surrounding the amyloid plaques. As a result, we observed that shAEBP1 injection induced the influx of microglia around Aβ plaques, and these microglia reduced the size of Aβ plaques (Figure 9). This suggests that regulation of AEBP1 in activated microglia is closely related to the phagocytosis of microglia, and further demonstrates the potential for developing therapeutics using regulation of AEBP1.

In conclusion, we confirmed that AEBP1 is a novel target molecule that can help in neuroprotection by regulating the immune response of activated microglia. Additionally, the downregulation of AEBP1 was shown to increase the responsiveness of microglia to pathogenic agents and to be closely related to phagocytosis. Although further validation of microglial phagocytosis in shAEBP1-injected mice and whether they can perform similar functions in other neurodegenerative disease models is required, our results are significant in suggesting the potential of AEBP1 for microglia-based diagnostics and therapeutics.

## 4. Materials and Methods

### 4.1. Cell Culture

BV2 microglial cells and SH-SY5Y neuronal cells were cultured in Dulbecco’s Modified Eagle Medium (DMEM)(Hyclone, Logan, UT, USA) supplemented with 10% fetal bovine serum (FBS)(Hyclone) and 1% penicillin G-streptomycin solution (PS)(Hyclone) at 37 °C in a 5% CO_2_ incubator.

### 4.2. RNA Extraction and Conventional Polymerase Chain Reaction (PCR)

RNA was extracted from BV2 cells using TRIzol reagent (Invitrogen, CA, USA) according to the manufacturer’s protocol. The extracted RNA was then converted into cDNA. cDNA synthesis was carried out using Diastar 2× RT pre-mix (Solgent, Daejeon, Republic of Korea), followed by purification using NanoDrop™ One (Thermo Fisher Scientific, Waltham, MA, USA) prior to use. Subsequently, conventional polymerase chain reaction (PCR) was performed using specific gene primers. Conventional PCR was performed according to the manufacturer’s instructions using Solg™ 2× Taq PCR Pre-Mix (Solgent). The PCR products were separated on 1% agarose gels (Molecular Biology Grade, Vivantis, Malaysia) by electrophoresis and visualized using a chemiluminescence analyzer (ViberLourmat, Eberhardzell, Germany). Gel images were obtained and analyzed using ImageJ software vesion 1.53. The primer sequences used for conventional PCR are summarized in Table 1.

### 4.3. Transfection of Short Hairpin RNA (shRNA) to BV2 Microglial Cells

Short hairpin RNA (shRNA), is a technology that suppresses the expression of specific genes through RNA interference (RNAi). This technique has the advantage of enabling sustained gene suppression within cells. In this study, shRNA was used to specifically suppress AEBP1 expression and analyze its function. In addition, to exclude nonspecific effects due to the transfection process itself, random shRNA (shScr) was used as the negative control.

shRNA of linear type produced by Genolution Pharmaceuticals (Seoul, Republic of Korea) was used in the experiment. The sequences for shRNA are listed in Table 2.

BV2 cells were seeded at $2\times 10^{5}$ cells/mL in a six-well plate. Afterwards, shRNA-AEBP1 and shScr were transfected using Lipofectamine 3000 (Invitrogen, Carlsbad, CA, USA) according to the manufacturer’s protocol. Four hours after transfection, the medium was changed to 10% DMEM, and the cells were cultured for 24 h before conducting the experiment.

### 4.4. Western Blot

Protein extraction from BV2 cells was performed using RIPA buffer. After harvesting, BV2 cells were washed twice with cold PBS. RIPA buffer was added, followed by a 15 min incubation on ice, and then centrifuged to obtain the supernatant for use in the experiment. The extracted proteins were quantified using the BCA method (Thermo Fisher Scientific, Waltham, MA, USA) according to the manufacturer’s instructions. Subsequently, gel loading was performed using a 10% running gel, followed by transfer onto a PVDF membrane. The membrane was washed with 1× TBS-T buffer and then incubated overnight at 4 °C with primary antibodies diluted in 3% skim milk: I*κ*Bα (1:1000) (#9242, Cell Signaling Technology (CST), Danvers, MA, USA), phospho-I*κ*Bα (1:1000) (#9241, CST), and β-actin (1:1000) (sc-4778, Santa Cruz, CA, USA). The following day, the membrane was washed with 1× TBS-T and incubated with a secondary antibody (1:10,000) (31430, Invitrogen, Carlsbad, CA, USA) conjugated with horseradish peroxidase (HRP) for 1 h. The membrane was then developed using an ECL buffer (Thermo Fisher), and the membrane image was captured using a chemiluminescence analyzer (ViberLourmat).

### 4.5. Enzyme-Linked Immunosorbent Assay (ELISA)

To investigate the secretion of the immune cytokine interleukin (IL)-6 by microglia with regulated AEBP1, BV2 cells were seeded in a 6-well plate and treated with LPS and shAEBP1. Culture supernatants were collected and subjected to ELISA (R&D Systems, Minneapolis, MN, USA) according to the manufacturer’s instructions. The assay was analyzed at 450 nm using a VersaMax microplate reader (Molecular Devices, Sunnyvale, CA, USA).

### 4.6. Flow Cytometry

BV2 cells treated with LPS or shRNA were prepared at a minimum of $1\times 10^{5}$ cells per 100 μL sample. The samples were then incubated with an MSR1 monoclonal antibody (1:200 dilution, E4H1C, CST) at 4°C for 2 h. Next, each sample was washed with 500 μL DPBS, and the secondary antibody (ab150077, Abcam, Cambridge, UK) was added to 100 μL DPBS and incubated at 4°C for 30 min. After the reaction, the cells were analyzed using flow cytometry (Novo Cyte Flow cytometry, ACEA Bioscience Inc., San Diego, CA, USA).

### 4.7. Phagocytosis Assay

To evaluate the phagocytic capacity of microglial cells, a phagocytosis assay was conducted using a Phagocytosis Assay Kit (IgG FITC) (Cayman Chemical, Ann Arbor, MI, USA). BV2 cells were seeded in a 6 well plate at a density of $2\times 10^{5}$ cells/mL and treated with LPS and shRNA to prepare the samples. Latex bead-rabbit IgG-FITC complexes were added to the pre-warmed culture medium at a final dilution of 1:500 and incubated at 37°C for 2 h. Following incubation, the cells were washed three times with assay buffer to remove background beads.

### 4.8. Cell Viability Assay

BV2 and SH-SY5Y cells were co-cultured in a 24-well plate (SPL Life Sciences, Gyeonggi-do, Republic of Korea) for the experiment. Before co-culture, BV2 cells were seeded in a 35 mm dish and transfected with shRNA. After transfection, SH-SY5Y cells were seeded in the lower well at $8\times 10^{4}$ cells/mL, and BV2 cells were seeded in the upper well at $6\times 10^{4}$ cells/mL. At 24 h post-cell seeding, the co-culture was performed, and an MTT assay was conducted 48 h later. For the MTT assay, 3-(4,5-dimethylthiazol-2-yl)-2,5-diphenyl tetrazolium bromide (MTT, Sigma, Poole, UK) solution (2 mg/mL) was added to the cells and incubated for 4 h. The formazan crystals were then dissolved in dimethyl sulfoxide (DMSO; Sigma-Aldrich, St. Louis, MO, USA) for 15 min, and the resulting solution was measured at 540 nm using a VerxaMax microplate reader (Molecular Devices).

### 4.9. Cell Apoptosis Assay

To evaluate the impact of AEBP1 knockdown in BV2 microglial cells on SH-SY5Y neuronal cell death, a cell apoptosis assay was conducted using an FITC-Annexin V Apoptosis Detection Kit (BD, Franklin Lakes, NJ, USA). A 12-well co-culture plate (Corning, NY, USA) was utilized, and BV2 cells previously treated with LPS or shRNA before co-culture were seeded at $8\times 10^{4}$ cells/mL in the upper well, and SH-SY5Y cells were seeded at $1\times 10^{5}$ cells/mL in the lower well. After 48 h, SH-SY5Y cells were harvested and washed twice with cold PBS. Subsequently, cells were resuspended in 1× binding buffer according to the manufacturer’s instructions, followed by incubation with 5 μL FITC-Annexin V and 5 μL Propidium iodide (PI) in the dark at room temperature for 15 min. An additional 1× binding buffer was added, and the samples were measured using flow cytometry (Novo Cyte Flow cytometry, ACEA Bioscience Inc., USA). Data analysis was performed using Novoexpress software vesion 1.2.5 (ACEA Bioscience Inc.).

### 4.10. Genomic DNA Extraction in Alzheimer’s Disease Mouse Model

The study utilized both normal wild-type (WT) mice and an Alzheimer’s disease model, 5× FAD mice. The frontal cortex (FC), hippocampal formation (HP), and olfactory bulb (OB) regions, known for their high density of microglial cells, were isolated from each mouse. Genomic DNA was extracted from these regions using the Exgene Genomic DNA micro kit (GeneAll, Seoul, Republic of Korea), following the manufacturer’s protocol. Conventional PCR was performed according to the manufacturer’s protocol. Gel electrophoresis was performed on 1% agarose gels (Molecular Biology Grade), and gel images were obtained using a chemiluminescence analyzer (ViberLourmat) for analysis in the study.

### 4.11. Animals

The 11-month-old transgenic mice with five familial AD mutations (5XFAD; Tg6799 Stock #006554; Jackson Laboratory, Bar Harbor, ME, USA) expressed mutations in human *PSEN1* and *APP* transgenes. *PSEN1* contains M146 and L286 mutations, while the *APP* transgene contains Florida (I716V), Swedish (K607Nand M671L), and London (V717I) mutations. These mice rapidly exhibit AD-related pathogenesis, including Aβ deposition, neurodegeneration, and cognitive dysfunction. Genotyping was performed by polymerase chain reaction analysis of tail DNA, and all experiments were blinded to the genotype. We used 11-month-old female 5XFAD mice for histological analysis of the frontal cortex. The experimental group was divided into two groups: (1) 5XFAD mice + vehicle (n = 5) and (2) 5XFAD mice + shAEBP1 (n = 5). The maintenance and treatment of animals in the experiments were in compliance with the principles of the Guide for the Care and Use of Laboratory Animals (National Institutes of Health publication No. 85-23, revised 1985) and the Animal Care and Use Guidelines of Konyang University (permit number: P-24-18-A-01).

### 4.12. Stereotaxic Injection of shAEBP1

Stereotaxic surgery was performed after aspiration anesthesia with isoflurane using an R500 Small Animal Anesthesia Machine (RWD Life Science Co., Ltd., Shenzhen, China). The 1.5 µg of shAEBP1 formulated with in vivo-jetPEI^®^ transfection reagent (Polyplus, Berkeley, CA, USA) was injected into the neocortical layer 5 at 0.5 µL/min for 3 min using a Hamilton syringe (26-gauge needle). Stereotaxic coordinates of cortical layer 5 were as follows: AP, +1.69 mm; ML, ±1.5 mm; DV, −2.0 and −2.2 mm from the bregma. The coordinates for stereotactic injection into cortical layer 5 were established and referenced from Paxinos and Franklin’s The Mouse Brain in Stereotaxic Coordinates. After the injection, the needle was carefully removed, and the skin was sutured.

### 4.13. Preparation of Brain Tissue

Seven days after shAEBP1 injections, the animals were anesthetized by intraperitoneal injection of Avertin (Tribromoethanol; Sigma-Aldrich, St. Louis, MO, USA) at a dose of 250 μg/kg. After anesthesia, the mice were transcardially perfused with 0.05 M phosphate-buffered saline (PBS) and then fixed with 4% paraformaldehyde (PFA) in 0.1 M phosphate buffer (PB). Next, the brains were collected, fixed in 4% PFA for 20 h at 4 °C, and immersed in 30% sucrose in PBS for cryoprotection. The brains were coronally sectioned at 30 µm thickness at −25°C using a cryostat (Leica Biosystems, Wetzlar, Germany). The sectioned tissues were stored at 4 °C in a cryoprotectant (0.05 M PB with 25% ethylene glycol and 25% glycerol) for histological analysis (Supplementary Figure S1).

### 4.14. Immunostaining

To investigate the effect of shAEBP1 on microglial recruitment in the brains of 5XFAD mice, two to three tissues were collected from the frontal cortex (between +1.42  and +2.22 mm from bregma; Figure S1). The free-floating brain slices were rinsed in PBS and incubated overnight in PBS containing 0.5 mg/mL bovine serum albumin and 0.3% Triton X-100 at 4 °C with the following primary antibodies: mouse anti-4G8 antibody (1:2000; BioLegend, San Diego, CA, USA) and goat anti-ionized calcium-binding adapter molecule 1 (Iba-1) antibody (1:500; Abcam, Cambridge, MA, USA). For staining with the 4G8 antibody, the brain tissues were incubated with 70% formic acid for 20 min for antigen retrieval before incubation with the primary antibody. After incubation with the primary antibody, the brain tissues were washed three times with PBS for 5 min and incubated for 50 min at room temperature with the following secondary antibodies: donkey Alexa 488-conjugated anti-mouse IgG and donkey Alexa 594-conjugated anti-goat IgG (1:300, Thermo Fisher Scientific) in 0.3% Triton X-100 containing PBS. Nuclei counterstaining and mounting were performed using Fluoroshield™ with DAPI (Sigma-Aldrich, St. Louis, MO, USA).

### 4.15. Image Acquisition and Analysis

Brain tissue images were acquired using a Zeiss LSM 700 (Carl Zeiss AG, Oberkochen, Germany) and analyzed using ImageJ software vesion 1.53 (NIH, Bethesda, MD, USA). We quantified the number of Iba-1 and DAPI double-labeled microglial cells around Aβ plaques to analyze the effect of shAEBP1 on microglial cell recruitment toward Aβ plaques. The average size of Aβ plaques was quantified in the same manner as described previously [40]. Image acquisition and analysis were performed blindly and randomly.

### 4.16. Statistical Analysis

All experiments were performed at least three times, and the measured data were calculated as mean ± standard error of the mean (SEM) and presented as a graph. The significance test between groups was analyzed using one-way ANOVA. Statistical differences are indicated in the figures. * *p* < 0.05, ** *p* < 0.02, *** *p* < 0.01. For statistical analysis, SPSS statistics software for Window, Version 18 (SPSS Inc., Chicago, IL, USA) was used. Statistical analyses of in vivo experiments were conducted using GraphPad Prism 10.0 software (GraphPad Software, La Jolla, CA, USA). Data are presented as mean ± standard deviation (SD). Differences between the two groups were analyzed using an independent *t*-test. Statistical difference was indicated by a *p*-value of <0.05, which exhibited a statistically significant.

## Figures and Tables

**Table 1:** Primer sequences used for PCR analysis.

Gene Name	Forward (5′ to 3′)	Reverse (5′ to 3′)
*GAPDH*	TCACCACCATGGAGAAGG	GCTAGGCAGTTGGTGGTGCA
*AEBP1-1*	GGGTGAGTACCGTGTGACAG	GGGTCAACTCGGAGAATGGG
*AEBP1-2*	GGAGACTGAGATGCCCACAC	GAGGTGGCTCGACTTCCTTC
*iNOS*	GAAGAAAACCCCTTGTGCTG	GTCGATGTCACATGCAGCTT
*TNF-* *α*	CAAGGGACAAGGCTGCCCCG	GCAGGGGCTCTTGACGGCAG
*IL-6*	ACAAGTCCGGAGAGGAGACT	GGTCTTGGTCCTTAGCCACTC
*ICAM1*	AGCACCTCCCCACCTACTTT	AGCTTGCACGACCCTTCTAA
*LCN2*	GCCCTGAGTGTCATGTGTCT	GAACTGATCGCTCCGGAAGT

**Table 2:** shRNA sequence for AEBP1.

shRNA Name	shRNA Sequence (5′ to 3′)
shRNA #1	CAGUUGUGGCCCGUUUCAU UCUC AUGAAACGGGCCACAACUG UU
shRNA #2	GCUACUACGCACAGAAUGA UCUC UCAUUCUGUGCGUAGUAGC UU
shRNA #3	ACAAUCACUCGCACAUACA UCUC UGUAUGUGCGAGUGAUUGU UU
shRNA #4	GCAAUGUGGACUACGAUAU UCUC AUAUCGUAGUCCACAUUGC UU
shRNA #5	GUGUCUUAUCCCUAUGACA UCUC UGUCAUAGGGAUAAGACAC UU
shScr	CCUCGUGCCGUUCCAUCAGGUAG UCUC CCUCGUGCCGUUCCAUCAGGUAG UU

## Data Availability

The original contributions presented in this study are included in the article/Supplementary Material. Further inquiries can be directed to the corresponding authors.

## Supplementary Materials

The following supporting information can be downloaded at: https://www.mdpi.com/article/10.3390/ijms27010296/s1.

## Abbreviations

The following abbreviations are used in this manuscript:

AEBP1	adipocyte enhancer-binding protein 1
AD	Alzheimer’s disease
Aβ	amyloid beta
FC	frontal cortex
HP	hippocampal formation
IL	interleukin
LPS	lipopolysaccharide
NFκB	nuclear factor-kappa-B
OB	olfactory bulb
SR-A1	scavenger receptor class A1
