# Supplementary material for: Targeting Adipocyte Enhancer-Binding Protein 1 to Induce Microglial Phenotype Shift for Immunotherapy in Alzheimer’s Disease
Source: Int J Mol Sci. 2025 Dec 27;27(1):296. doi: 10.3390/ijms27010296 (PMC12785470; doi:10.3390/ijms27010296)
Supplement: Supplementary file 1 [file ijms-27-00296-s001.zip › ijms-4021371-supplementary.pptx]

## Slide 1
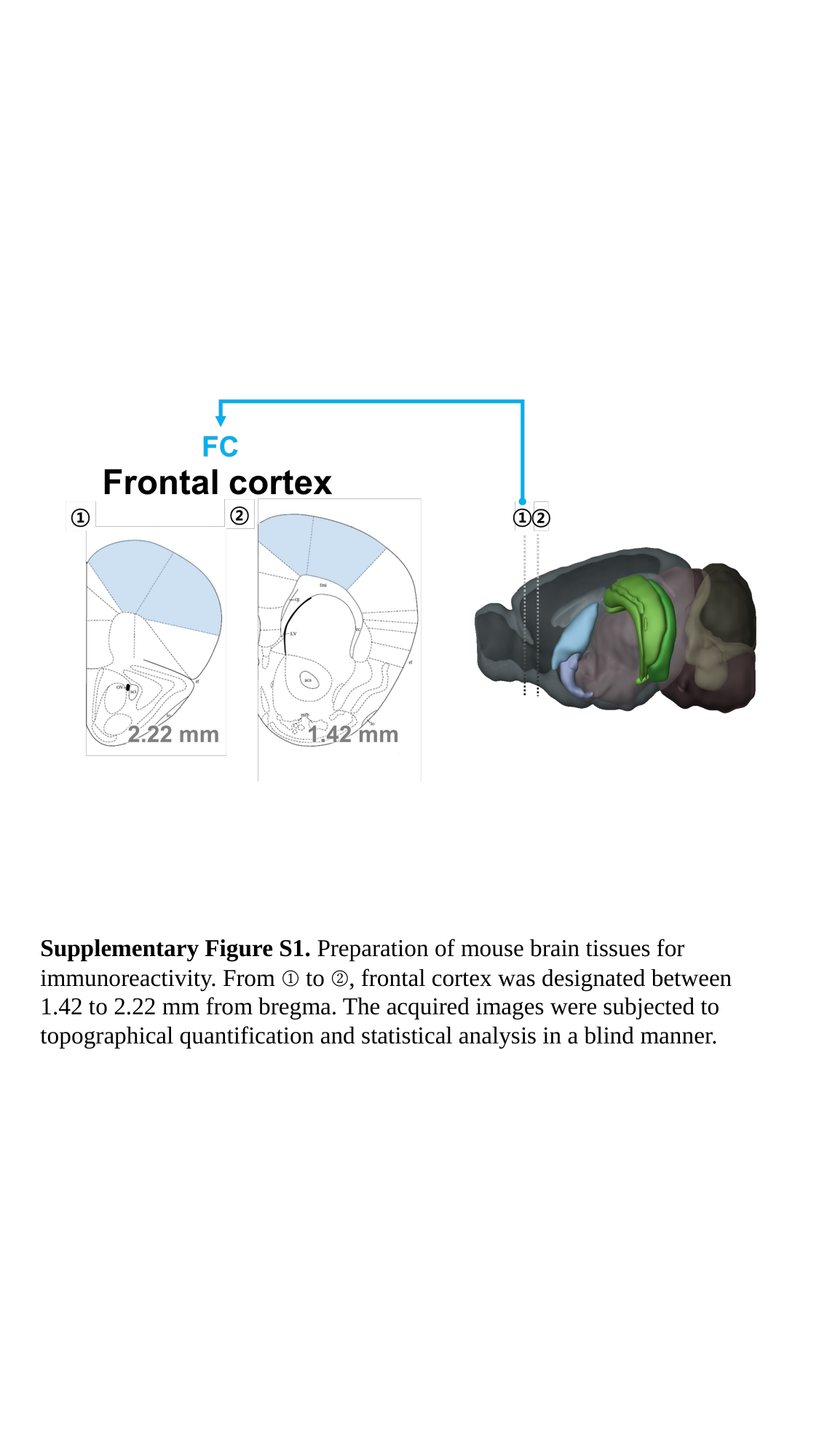

Supplementary Figure S1. Preparation of mouse brain tissues for immunoreactivity. From ① to ②, frontal cortex was designated between 1.42 to 2.22 mm from bregma. The acquired images were subjected to topographical quantification and statistical analysis in a blind manner.
